# An accurate green fruits detection method based on optimized YOLOX-m

**DOI:** 10.3389/fpls.2023.1187734

**Published:** 2023-05-08

**Authors:** Weikuan Jia, Ying Xu, Yuqi Lu, Xiang Yin, Ningning Pan, Ru Jiang, Xinting Ge

**Affiliations:** ^1^ School of Information Science and Engineering, Shandong Normal University, Jinan, China; ^2^ School of Information Science and Engineering, Zaozhuang University, Zaozhuang, China; ^3^ School of Agricultural Engineering and Food Science, Shandong University of Technology, Zibo, Shandong, China; ^4^ School of Medical Imaging, Xuzhou Medical University, Xuzhou, China

**Keywords:** green fruits, YOLOX_m, Atrous spatial pyramid pooling, varifocal loss, object detection (OD)

## Abstract

Fruit detection and recognition has an important impact on fruit and vegetable harvesting, yield prediction and growth information monitoring in the automation process of modern agriculture, and the actual complex environment of orchards poses some challenges for accurate fruit detection. In order to achieve accurate detection of green fruits in complex orchard environments, this paper proposes an accurate object detection method for green fruits based on optimized YOLOX_m. First, the model extracts features from the input image using the CSPDarkNet backbone network to obtain three effective feature layers at different scales. Then, these effective feature layers are fed into the feature fusion pyramid network for enhanced feature extraction, which combines feature information from different scales, and in this process, the Atrous spatial pyramid pooling (ASPP) module is used to increase the receptive field and enhance the network’s ability to obtain multi-scale contextual information. Finally, the fused features are fed into the head prediction network for classification prediction and regression prediction. In addition, Varifocal loss is used to mitigate the negative impact of unbalanced distribution of positive and negative samples to obtain higher precision. The experimental results show that the model in this paper has improved on both apple and persimmon datasets, with the average precision (AP) reaching 64.3% and 74.7%, respectively. Compared with other models commonly used for detection, the model approach in this study has a higher average precision and has improved in other performance metrics, which can provide a reference for the detection of other fruits and vegetables.

## 1 Introduction

In the world, the annual consumption of fruits in all countries is huge and has been showing an increasing trend, so the production and planting area have been expanding in recent years, which requires a lot of human resources. In order to reduce labor costs, the production and management of modern agriculture is gradually developing in the direction of automation. In recent years, computer vision technology is gradually being applied to modern agriculture because of its role in vision systems for agricultural automation equipment, such as pest and disease identification and detection (He et al., 2013; Fuentes et al., 2017; Johnson et al., 2021), automated harvesting of fruits and vegetables (Jia et al., 2020; Wang et al., 2022; Tang et al., 2023), crop growth information monitoring and yield estimation (Apolo-Apolo et al., 2020; Li et al., 2020), and so on. The precision of the vision system detection determines the efficiency of the automated equipment, and the complexity of the modern orchard environment makes its ability to accurately detect the target fruit dependent on a variety of factors, such as the angle of light, weather conditions, and the overlap of shading between fruits, etc. In addition, the color of most immature fruits is green, so the research on the detection of green fruits is important for the subsequent operation of fruits, such as yield estimation and fruit harvesting, etc., but the similar color of immature green fruits and leaves will cause the boundary to be more difficult to distinguish, which will also have an impact on the precise detection of fruits. These problems have attracted the attention of many domestic and international scholars, who have carried out some relevant research and achieved some results.

Traditional machine learning plays an important role in the field of computer vision, and many results have been achieved in machine learning detection research in agricultural fruit detection. Linker (Linker et al., 2012) proposed a green apple recognition model based on fruit characterization information with a correct detection rate close to 95%. Wu (Wu et al., 2020) proposed a fruit point cloud segmentation method combining color and 3D geometric features, where local descriptors were used to obtain candidate regions and global descriptors were used to obtain the final segmentation results. Wang (Wang et al., 2021) proposed a new kernel density clustering (KDC) to better realize the accurate identification of green apples. Tian (Tian Y. et al., 2019) proposed a fruit localization algorithm based on image depth information, which fits the detection region by introducing a segmentation algorithm to locate the center and radius of the apple circle, respectively, through the gradient information obtained from the depth apple image and the corresponding RGB spatial information. Moallem (Moallem et al., 2017) used the multilayer perceptron (MLP) and k-nearest neighbors (KNN) to classify the apples with 92.5% and 89.2% recognition rates for the extracted features, respectively. Traditional machine learning for agricultural fruit detection is relatively well established, but the limitations of machine learning also limit the speed and precision of object detection.

In recent years, with the rapid development of deep learning and convolutional networks, they have eliminated some of the limitations and complex operations of traditional machine learning. Computer vision has also shifted its research focus to deep learning and convolutional networks, and has been widely used in many fields. At present, research on vision systems for agricultural automation equipment has also focused on deep learning models, and some results have been achieved. Sun (Sun et al., 2022) proposed a balanced feature pyramid for small apple detection, which achieved an average detection accuracy of 35.6% for small targets on the Pascal VOC benchmark with good generalization performance. Wang (Wang and He, 2019) proposed an apple object detection and recognition algorithm based on R-FCN. The model uses ResNet-44 as the backbone network, which improves the detection accuracy and simplifies the network. Triki (Triki et al., 2021) proposed a Mask RCNN-based leaf detection and pixel segmentation technique that can segment leaves of different families, measure the length and width of leaves, and reduce the recognition error. Mu (Mu et al., 2020) performed detection of highly shaded unripe tomatoes based on deep learning techniques, combined with regional convolutional networks (R-CNN) and Resnet-101, for ripeness detection and yield prediction of tomatoes. Jia (Jia et al., 2022b) proposed a Mask R-CNN based segmentation model RS-Net, which achieves robust segmentation of green apples to meet the accuracy and robustness of vision systems in agronomic management. Kang (Kang and Chen, 2020) obtained DASNet-v2 by improving DASNet, which uses visual sensors to segment apple instances, so it can achieve segmentation of fruits more robustly and efficiently.

Compared with traditional machine learning, the detection accuracy of the above research has been greatly improved, but due to the complex environment of real orchards, the existence of difficult detection conditions such as leaves obscuring fruits, overlapping fruits, and the similar color of fruits and branches, the accuracy of the above methods for fruit detection still does not meet the needs of modern automated agriculture, and the precision needs to be further improved.

Therefore, in order to simulate the actual environment of the orchard as much as possible, this paper collects images of green apples and persimmons in various complex situations to make two datasets and proposes an improved YOLOX-m network model to improve the detection accuracy of the fruits. The model uses the CSPDarknet backbone network to better extract image features. In the multi-scale feature fusion stage, referring to the PAnet structure, it will not only upsample the features to achieve feature fusion, but the features are also downsampled to achieve feature fusion, and ASPP (Atrous spatial pyramid pooling) is used to increase the receptive field during fusion, so that each convolution output contains a larger range of information, thereby improving network performance and reducing the rate of missed and wrong detections. In addition, Varifocal loss is used instead of BCE (binary cross-entropy) loss to mitigate the negative effects of sample imbalance and better optimize the model parameters to improve the detection accuracy of green fruits in complex orchard environments.

## 2 Datasets production and experimental setup

### 2.1 Datasets collection

**Figure 1:**
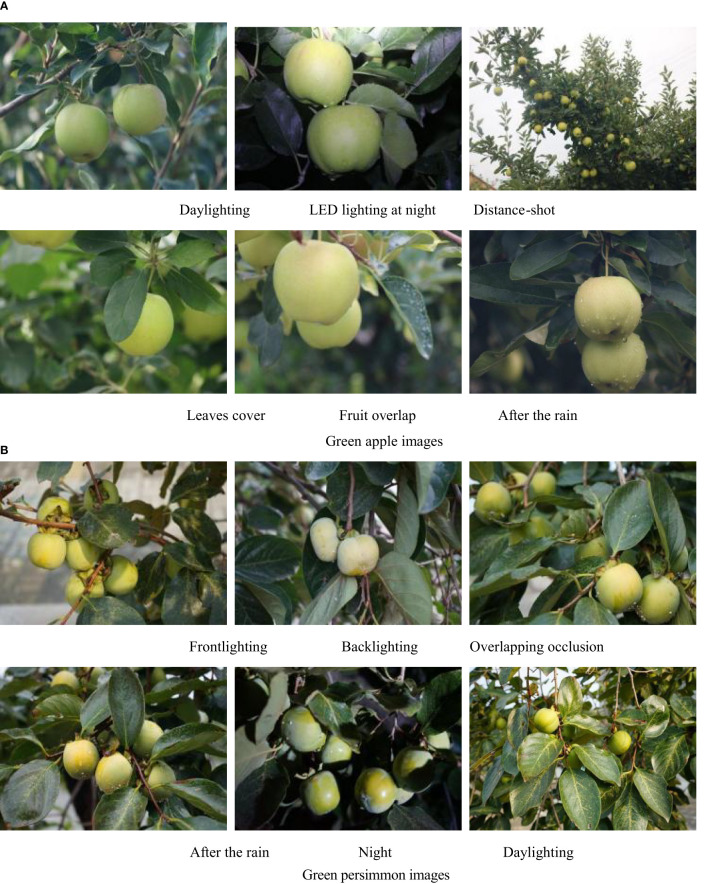
Images of green fruit in different situations. **(A)** Green apple images **(B)** Green persimmon images.

The datasets used in this paper are the immature green persimmon and green apple datasets. The persimmon images constituting the dataset were collected from the back mountains of Shandong Normal University (Changqing Lake Campus) and the southern mountainous region of Jinan, using a Canon EOS 80D SLR camera with a CMOS image sensor, and the apple images were collected from the apple production base in Longwang Mountain, Fushan District, Yantai City, Shandong Province, using a Sony Alpha 7 II camera. The image resolution was 6000 pixels × 4000 pixels, saved in.jpg format, and 24-bit color image. **Figure 1** list several collected images of apples and persimmons in different complex situations.

The actual environment of the orchard is more complex, and in order to simulate the real situation as much as possible, the dataset collects images of different complex situations. It is not easy to discriminate overlapping fruit boundaries by shading, water drops on fruits after rain can be a factor affecting detection, and different lighting can also affect the final detection effect. Considering, a total of 553 images of green persimmons and 1361 images of green apples were finally collected under different situations, including down-lighting, back-lighting, daytime and nighttime LED lighting, overlapping fruits, and leaf shading. Among them, the persimmon and apple datasets contained 2524 and 7137 fruits, respectively, and **Table 1** shows the number and proportion of fruits of different scale sizes, where the ground truth box area less than $32^{2}$ belongs to the small-scale target fruits, the ground truth box area between $32^{2}\text{ and }96^{2}$ belongs to the medium-scale target fruits, and the ground truth box area greater than $96^{2}$ belongs to the large-scale target fruits.

**Table 1:** The divided results of datasets by area size of fruit.

Area	Small	Medium	Large	Fruit Total	Image Total
Apple Dateset					
Train	1701/34%	2007/41%	1235/25%	4943	953
Val	851/39%	816/37%	527/24%	2194	408
Total	2552/36%	2823/39%	1762/25%	7137	1361
Persimmon Dataset					
Train	272/15%	1111/59%	482/26%	1865	388
Val	47/7%	415/63%	197/30%	659	165
Total	319/13%	1256/60%	679/27%	2524	553

### 2.2 Datasets production

The collected apple and persimmon images were divided into training set and test set in the ratio of 7:3. After the division, the apple training set included 953 images and the test set included 408 images; the persimmon training set included 388 images and the test set included 165 images. And in order to reduce the computational effort and the subsequent experiment time, the image resolution was uniformly scaled from 6000×4000 pixels to 600×400 pixels. The labeling software used is LabelMe, and the edge contours of the fruit are labeled with labeling points, so that the fruit can be separated from the background. The labeling information of the image and the coordinates of the labeling points are saved in the corresponding.json file, and the completed json file is finally converted into a coco format dataset (Lin et al., 2014).

## 3 Optimized YOLOX-m network

**Figure 2:**
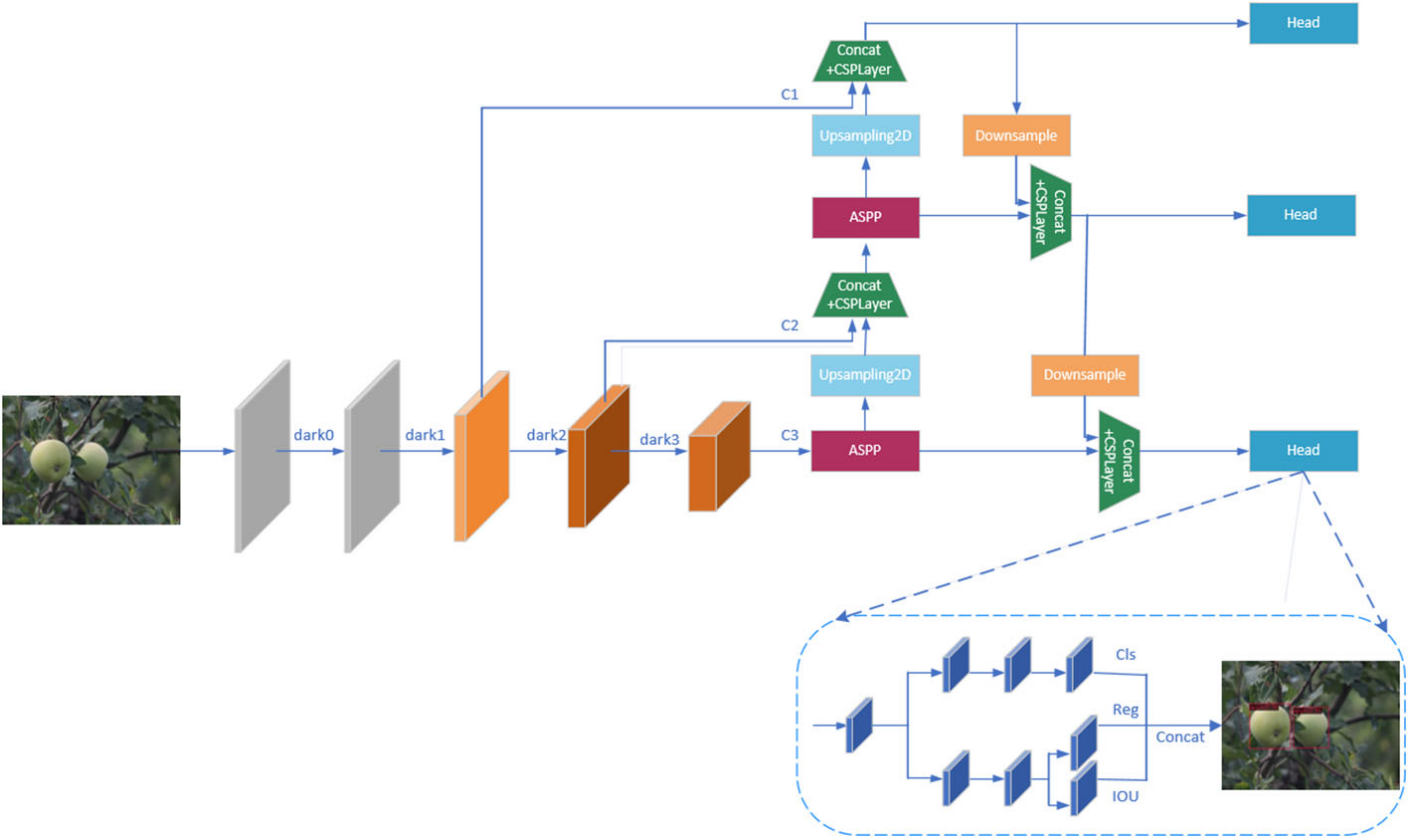
Improve YOLOX-m network detection framework.

The actual orchard environment is complex and variable, and the color of green fruits is similar to the leaves, which further makes the boundary between the background and the fruits blurred and unclear, not easy to decide, causing the detection of fruits to be more difficult and affecting the final accuracy of detection. In order to improve the object detection accuracy of green fruits and improve the vision system of agricultural automation equipment, this paper proposes an improved YOLOX_m (Ge et al., 2021) model for efficient detection of green fruits, and the specific detection framework is shown in **Figure 2**.

The model in this paper uses CSPDarknet (Bochkovskiy et al., 2020) as the backbone network for feature extraction of apple and persimmon images, and the input images will get three effective feature layers C1, C2, and C3 through the backbone network. The bottom feature layer has less semantic information, but accurate target location information, and the higher-level features have rich semantic information, but locate the target location more roughly, so the feature map needs to go through a feature fusion pyramid (Lin et al., 2017a) for feature fusion before classification and regression prediction, combining feature information of different scales. As shown in **Figure 2**, in the feature fusion stage, the model in this paper introduces the Atrous Spatial Pooling Pyramid (ASPP) module before the upsampling operation, which sets different dilation rates to construct convolution kernels with different receptive fields, and increases the receptive fields by parallelizing multiple Atrous convolution layers with different dilation rates to obtain multi-scale information of the target, so as to more effectively enhance feature extraction and improve the detection accuracy of the target green fruits. The three fused feature layers are input to the prediction head, and the prediction head of the model is decoupled to perform classification and regression to determine whether the target is a green fruit or a background, and to accurately locate the target fruit.

In addition, although the original model reduces the number of negative samples, the target fruit still only accounts for a small portion of the entire input image, and the number of positive samples is still far less than the number of negative samples. To further alleviate the negative impact of sample imbalance, the loss function was replaced from BCE (binary cross-entropy) loss to Varifocal loss (Zhang et al., 2021) to make the model focus more on difficult to classify samples and to focus training on positive samples, which can better optimize the model parameters, improve detection accuracy and reduce the false detection rate, thus improving the fruit picking and yield prediction and other aspects of accuracy.

### 3.1 Backbone network CSPDarkNet

**Figure 3:**
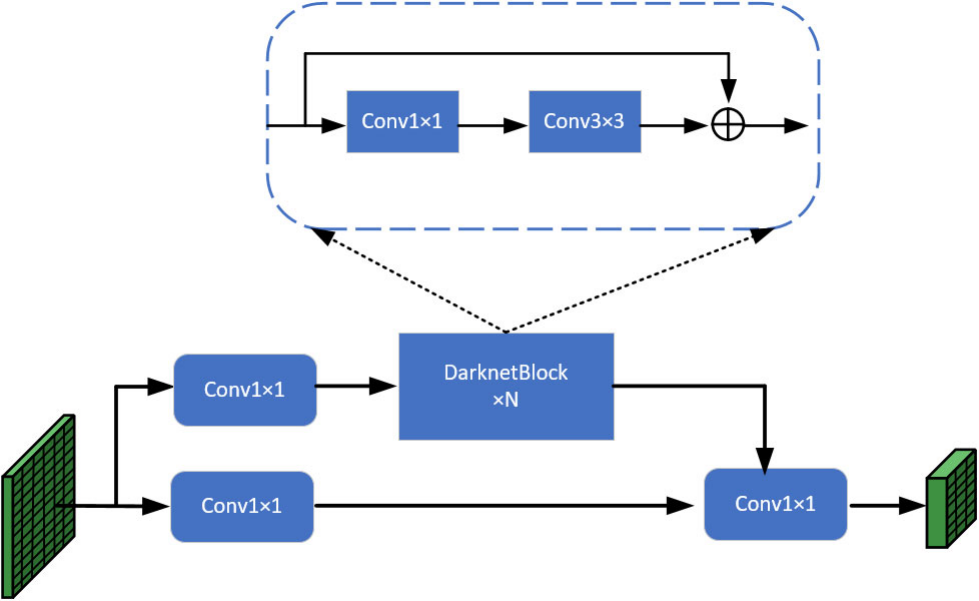
CSPLayer structure schematic diagram.

Taking into account the difficult detection problems such as the similarity of green fruits to the background and the overlapping of fruit occlusion, in order to extract the features of the images more effectively, the model in this paper uses CSPDarkNet as the backbone network, and the input immature green persimmon and green apple images use the backbone network CSPDarkNet for feature extraction to obtain three effective feature layers of different scales, using them for subsequent training and prediction. The residual module in CSPDarkNet is based on the network structure of residual network and CSPNet (Wang et al., 2020). The jump link in the residual network can effectively mitigate the gradient disappearance problem as the network depth increases, while the use of CSP structure can enhance the learning ability of the convolutional neural network and speed up the inference. First, the input image is passed through the Fcous network to reduce the number of parameters and improve the running speed of the model, then, after a series of operations of convolutional regularization and activation function for a channel expansion, and finally, three effective feature layers of different scales are output in turn through four residual modules, and the structure of CSPLayer in the residual module is shown in **Figure 3**.

The green persimmon and apple images are continuously feature extracted by four residual modules in the backbone network CSPDarkNet. During this process, the width and height of the feature maps are continuously halved and the number of channels is expanded to twice. When passing through the last three residual modules, three effective feature layers at different scales are output respectively. Although the semantic information is gradually enriched during the feature extraction process, the image resolution decreases and the boundary information is lost, so the information contained in the three feature layers will be different. Therefore, before inputting the feature map into the head for prediction, it is necessary to fuse the features of different scales through the feature pyramid, so as to better predict the fruit for classification regression.

### 3.2 Feature pyramid network

**Figure 4:**
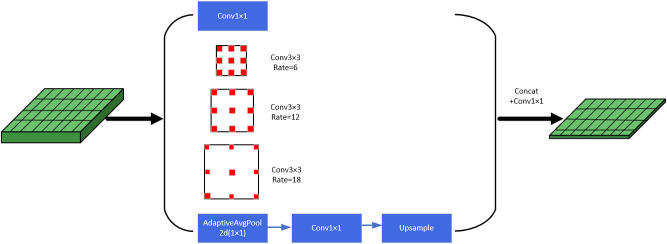
Atrous spatial pyramid pooling structure.

Originally, Atrous convolution and ASPP (Atrous Spatial Pyramid Pooling) (Sullivan and Lu, 2007) were proposed in the semantic segmentation model DeepLabv2 (Chen et al., 2017). Compared with ordinary convolution, atrous convolution has an additional parameter dilation rate, which increases the receptive field of the convolution kernel without causing information loss. Atrous convolution is an important part of the ASPP module, which sets different dilation rates to construct convolution kernels with different receptive fields, and obtains multi-scale information of the target by parallelizing multiple atrous convolution layers with different dilation rates. In this way, the receptive field can be increased while ensuring that there is not much loss of resolution. If the loss of resolution is too large, the information of the fruit image boundary will be lost, which is not beneficial to the detection of green fruits. The module specifically consists of a 1×1 convolution, three atrous convolution layers with different dilation rates, and an atrous pooling layer in parallel, and the obtained results are concatenated in the channel dimension, and then, the output is obtained after another 1×1 convolution layer for channel number reduction. The specific structure as shown in **Figure 4**.

When feature extraction is performed on apple and persimmon images, the semantic information and location detail information of the feature layers change continuously because of the need for constant convolution and down sampling. The initial low-level feature layer C1 is rich in spatial information and locates the location more accurately, but contains less semantic information, so the green fruits with similar color to the branches and leaves are more difficult to be determined and easy to detect incorrectly. The feature layer from C1 to C3, the semantic information becomes richer, but the resolution gradually decreases and the detail information such as boundary is lost, so the localization of the target fruit is rougher, and the higher feature layer C3 can determine the target species more accurately, but it is not conducive to the localization of the target fruit. Therefore, to improve the accuracy of final classification and localization, a feature pyramid network is used to enhance feature extraction, and the feature layers of different scales of green fruit images are complemented with advantages to make the information of feature layers more comprehensive. The feature fusion pyramid network used in this model refers to the structure of PANet (Liu et al., 2018). In the process of feature fusion, it will not only start from the high level features, perform up-sampling operation and fuse with the low level features, but also perform down-sampling operation on the three feature layers after up-sampling and fusion from the low level, and perform feature fusion again to get the final input head for prediction of feature layers.

Before the upsampling operation, the feature layer needs to be down-dimensioned by a 1×1 convolution to reduce the number of channels. In order to increase the receptive field, capture multi-scale information, and better extract features at different scales, the model in this paper adds a 1×1 convolution layer, atrous convolution layers with different dilation rates, an atrous pooling layer, etc. in parallel before the dimensionality reduction operation, and concatenates the results together. Therefore, Atrous Spatial Pyramid Pooling (ASPP) is introduced to replace the 1×1 convolutional layer before upsampling to obtain more accurate localization and classification information of the target green fruits.

### 3.3 Loss function

The construction of the loss function has an important significance to the training of the model, and the main role is that during training, the model will use the loss values obtained during forward propagation to update the training parameter weights through backward propagation. After continuous iterations, the loss difference between the prediction box and the ground truth box is gradually reduced, and the loss function will gradually reach the minimum value, so that the prediction box gradually overlaps close to the ground truth box, thus achieving accurate localization of the target green fruits. In this paper, the loss of the model during training mainly contains classification loss, regression loss and confidence loss. The IOU (intersection of union) loss is used for the regression loss, and the Varifocal loss is used for the classification and confidence loss, and the formula for the overall loss function of the model is shown in equation (1).


(1) $$\text{Loss }=\frac{1}{{\text{N}}_{\text{pos}}}\left({\text{L}}_{\text{cls}}+\lambda L_{reg}+L_{obj}\right)$$

Where *N*_pos_ refers to the number of feature points that are assigned as positive sample points, $L_{cls}$ refers to the classification loss, $L_{\text{reg}}$ refers to the regression loss, and $L_{\text{obj}}$ refers to the confidence loss, λ is the balance coefficient of the regression loss, set to 5.0.

The regression loss refers to the IOU loss between the ground truth box and the predicted box, and is calculated as shown in equation (2).


(2) $$\text{IOU loss }=\;-\text{ln}\frac{\text{Intersection}\left({\text{B}}_{\text{gt}},{\text{B}}_{\text{pred}}\right)}{\text{Union}\left({\text{B}}_{\text{gt}},{\text{B}}_{\text{pred}}\right)}$$

where $\text{Intersection}\left(B_{gt},B_{pred}\right)$ refers to the area where the real frame intersects the prediction frame, and $\text{Union}\left(B_{\text{gt}},B_{pred}\right)$ refers to the area where the real frame and the prediction frame are combined and summed.

In the actual training phase of the model, the target green fruit only accounts for a small portion of the whole input image, so the number of negative samples is much larger than the number of positive samples, and there will be an unbalanced distribution of positive and negative samples, which will lead to a decrease in training accuracy and the optimization direction of the model is not as desired. In addition, the commonly used loss function BCE loss does not distinguish between samples that are difficult to classify and those that are easy to classify. When the negative samples that are easy to classify are much more than the positive samples, the model will focus more on these negative samples and drown out the impact of the positive samples that help training, causing a loss in the final detection precision. In order to alleviate the above negative effects and improve the detection accuracy of fruits, the classification and confidence loss function of the model in this paper adopts Varifocal loss, which is based on BCE loss, and the specific formula of the loss function is shown in equation (3).


(3) $$\text{VFL}\left(\text{p},\text{q}\right)=\{\begin{array}{c} -q(qlog\left(p\right)+\left(1-q\right)log\left(1-p\right)\;\;q>0\\ -\alpha {\text{p}}^{\gamma }log\left(1-p\right)\;\;\;\;\;\;\;\;\;\;\;\;\;\;\;\;\;\;q=0 \end{array}$$

In the formula, α,γ are hyperparameters, α is the balance parameter to adjust the weight of positive and negative samples, and the tempering factor ${\text{p}}^{\gamma }$ can reduce the influence of easy to classify samples on the loss and make the model focus more on difficult to classify samples, such as targets in the image that are obscured by leaves or overlapped with other fruits. Varifocal loss is treated differently for positive and negative samples compared to focal loss. For negative samples, q=0, in this case, ${\text{p}}^{\gamma }$ can be used to reduce the loss contribution of negative samples, and for positive samples, which is the case of q>0, the value of q is the IOU between the prediction box and the ground truth box, and q is used to weight the positive samples, so that when the positive sample has a higher IOU, its contribution to the loss is also large, and it allows the model to focus its training on high-quality positive samples, which can result in better detection accuracy and better detection of the target green fruits.

## 4 Experiments

### 4.1 Experimental design and operation platform

The server environment used for model training in this paper is Ubuntu 18.04 OS, NVIDIA A30 graphics card and 11.1 CUDA environment. The programming language used in the model is python, and the Pytorch 1.8 (Paszke et al., 2019) deep learning library is also used in this process, and the implementation is built with the help of MMdetection (Chen et al., 2019) related modules.

**Figure 5:**
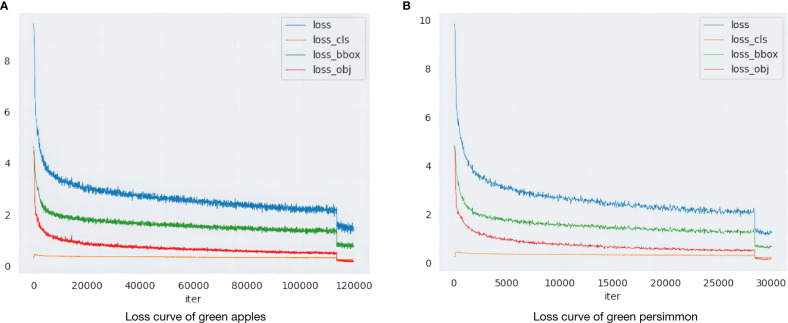
Loss function change curve. **(A)** Loss curve of green apples. **(B)** Loss curve of green persimmon.

Before formal training, the pre-training weights obtained using the ImageNet dataset are imported as initialization parameters to accelerate the detection speed and improve the robustness of the model. In the formal training phase, the model parameters are optimized and updated using the SGD optimizer. The learning rate, momentum factor, and weight decay factor are set to 0.00125, 0.9, and 0.0005, respectively, and 300 epochs are trained iteratively, and the parameter results are saved once every 10 iterations. The variation of the loss during training is shown in **Figures 5A, B**, where the x-axis represents the number of iterations and the y-axis indicates the value of the loss function, and different colors are used to distinguish the various types of losses.

### 4.2 Assessment metrics

In order to comprehensively evaluate the performance of the model, this paper uses a variety of assessment metrics to evaluate the effect, among which the main consideration is the average precision (AP) of detection. The precision (P) is the probability of the samples being correctly predicted among all samples, calculated as shown in equation (4), and the recall (R) is the probability of the positive samples being correctly predicted among the prediction results, calculated as shown in equation (5).


(4) $$P=\frac{\text{TP}}{\text{TP}+FP}$$

(5) $$R=\frac{\text{TP}}{TP+FN}$$

Where TP, FP, and FN are the number of true positive samples, the number of false positive samples, and the number of false negative samples, respectively. Further it is possible to calculate the AP (Average precision) under a specific IOU threshold, and the calculation formula is shown in equation (6).


(6) $${\text{AP}}^{\text{IOU}=i}=1/101\sum_{r\in R}\underset{\tilde{r}:\tilde{r}\geq \text{r}}{\max p(}\tilde{r})$$

where *i* is the value of the settable IOU threshold, whose value can be set in a range greater than or equal to 0.5 less than 1, i ∈ I[0.5,0.55,0.6, ……, 0.95], with a total of 10 values, p(r) denotes the accuracy rate associated with the recall, R ∈ [0, 0.01, 0.02, ……, 1] with 101 values, and r denotes the value taken as the recall rate. Continuing to average the 10, the final AP metric used can be obtained, and the formula is shown in equation (7).


(7) $$AP=\frac{1}{10}\overset{\sum }{i\in I}AP^{IOU=i}$$

In order to evaluate the performance of the model approach in more detail, a number of other evaluation metrics are used. AR refers to the average recall; $AP^{IOU=0.5}$ and $AP^{IOU=0.75}$ refer to the AP value when the IOU threshold is over 0.5 and 0.75, respectively; ${\text{AP}}_{S}$ , ${\text{AP}}_{M}{\text{ and AP}}_{L}$ refer to the average detection accuracy for small, medium and large scale target fruits, respectively, where the ground truth box area less than $32^{2}$ belongs to the small-scale target fruits, the ground truth box area between $32^{2}\text{ and }96^{2}$ belongs to the medium-scale target fruits, and the ground truth box area greater than $96^{2}$ belongs to the large-scale target fruits; In addition, Time refers to the speed of validation set detection to evaluate an image in *ms*; Params refers to the total parameters to measure the size of the model; and FLOPs refers to floating point operations to measure the computational complexity of the model.

### 4.3 Results and analysis

#### 4.3.1 Green fruit detection effect

**Figure 6:**
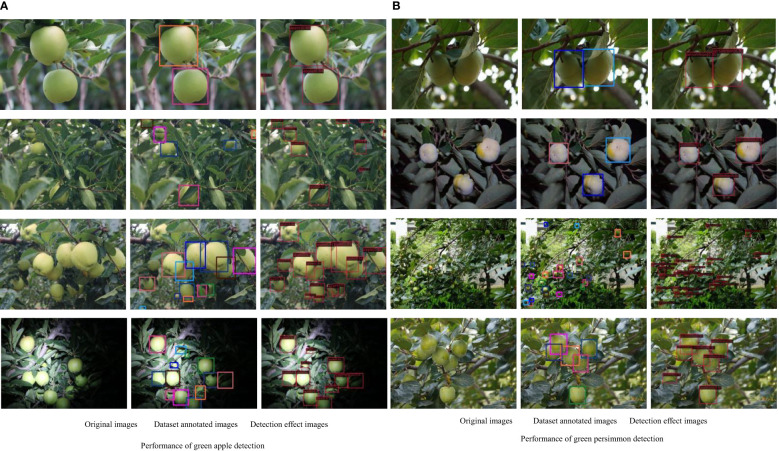
Green fruits detection effect images. **(A)** Performance of green apple detection **(B)** Performance of green persimmon detection.

In this paper, we use the improved yolox_m network model to analyze the target fruit detection effect on the collected immature green persimmon and green apple datasets. The pictures contained in the datasets restore the complex environmental conditions of real orchards as much as possible, considering different shooting distances, different situations such as overlapping fruit shading, after rain, at night and smooth backlighting, etc. The detection effect under several situations is selected for analysis, and the specific detection effect is shown in **Figures 6A, B**.

As can be seen from **Figure 6**, we can see that the sparse and independent fruits will have a clearer and more complete outline, so the detection accuracy of such target fruits is better, and the detection effect of the images collected at night can also reach a better level. In terms of distance, the detection effect of close-range fruit is better than that of distant target fruit. For those densely-distanced fruit or occluded and overlapping target fruit, the detection is relatively difficult, and the accuracy is slightly reduced, but there are almost no omissions and errors.

**Figure 7:**
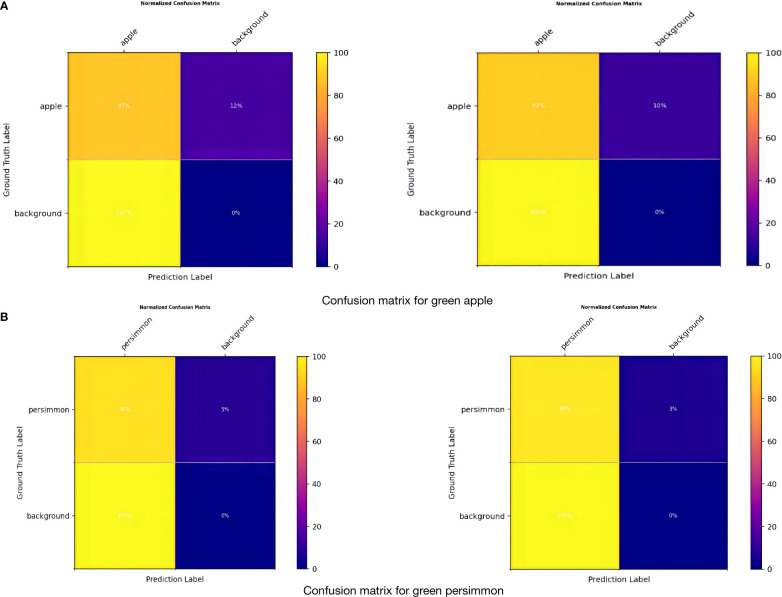
Confusion matrix. **(A)** Confusion matrix for green apple. **(B)** Confusion matrix for green persimmon.

In **Figures 7A, B**, it can be seen that True Positive is 89% and 96% for apples and persimmons, respectively, which is an improvement of 2%, and False Negative is a decrease of 2%. Overall, although the complex reality of orchards brings some negative effects on detection, the model in this paper achieves a good level of detection accuracy for target fruits, and some target fruits that were not labeled at the time of dataset labeling can be detected, with a decrease in the rate of omission and error.

**Figure 8:**
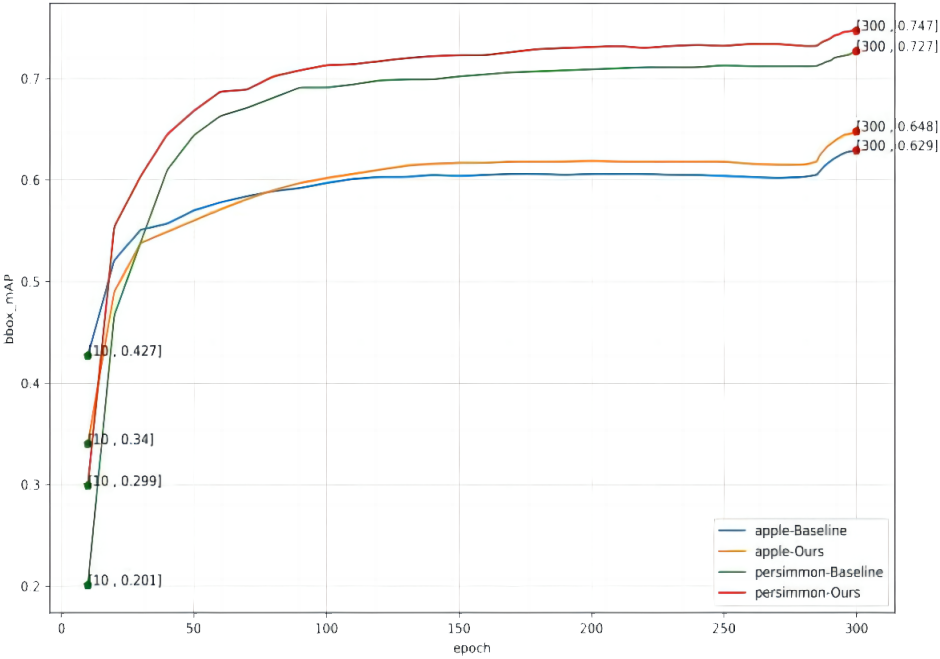
mAP curve for each epoch.

In order to fully verify the performance of the model in this paper, the model was tested on two datasets, apple and persimmon, and the model detection effect basically reached the highest accuracy after the last epoch. In order to validate the improvement effect, the original network without improvement is recorded as yolox_origin, the network with only the improved feature pyramid is recorded as yolox_A, the network with only the improved loss function is recorded as yolox_V, and the network with all the improvements is recorded as yolox_after, and the results of various evaluation indicators on the validation set are shown in **Table 2**, and the change curve of mAP is shown in **Figure 8**.

**Table 2:** Image detection and evaluation results.

Network	Metric						
	AP	*AP^IOU^* ^=0.5^	*AP^IOU^* ^=0.75^	AP_S_	*AP_M_*	*AP_L_*	AR
Apple Dataset	%						
yolox_origin	62.9	87.3	68.4	44.3	69.4	91.9	68.6
yolox_A	63.7	88	70	46.6	69.8	90.9	69.7
yolox_V	63.8	87.4	69.8	46.4	70.2	91.4	69.5
yolox_after	64.8	88.4	71.2	47.7	70.7	92.1	72.6
Persimmon Dataset	%						
yolox_origin	72.7	91.3	82.1	36.6	73.9	86.7	78.5
yolox_A	74	91.6	84.6	39.2	74.8	88.2	79.6
yolox_V	73.6	91.5	83.3	36.6	74.5	88.3	80.5
yolox_after	74.7	91.9	84	39	75.6	89.4	81.5

From **Table 2** and **Figure 8**, it can be found that the final detection average precision of the method in this paper for green apple and green persimmon images is 64.8% and 74.7%, and the average recall rate is 72.6% and 81.5%, respectively. It can be seen from the table that using the atrous spatial convolution pooling pyramid (ASPP) and the loss function using the Varifocal loss can improve the detection accuracy of the model and improve the model performance on both datasets. In addition, $AP^{IOU=0.5}\text{ and }AP^{IOU=0.75}$ have also been greatly improved, and in both data sets, the average accuracy of large, medium and small targets has been improved to a certain extent. The detection accuracy on large targets can also reach about 90%.

#### 4.3.2 Comparison of model detection effects

**Figure 9:**
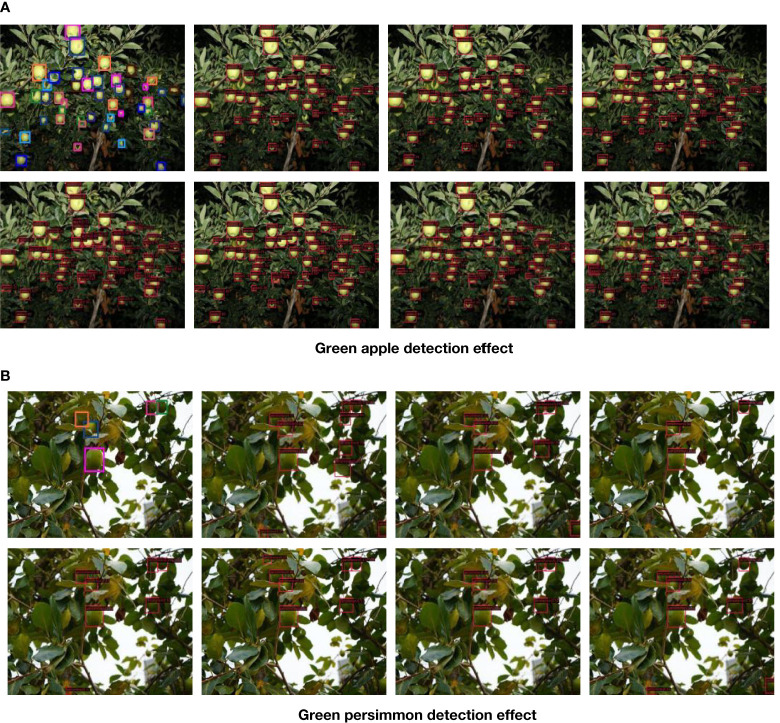
Different model detection effect. **(A)** Green apple detection effect. **(B)** Green persimmon detection effect.

In order to objectively analyze and compare the performance of the model in this paper, we compare the model with several common and representative object detection model algorithms. The selected models are FCOS (Tian et al., 2019), Faster-RCNN (Ren et al., 2015), YOLOv3 (Redmon and Farhadi, 2018), SSD (Liu et al., 2016), FSAF (Zhu et al., 2019) and ATSS (Zhang et al., 2020), where Faster-RCNN is a two-stage detection model based on anchor frames, YOLOv3, SSD and ATSS are single-stage detection models based on anchor frames, and FCOS as well as FSAF belong to the detection model with anchor-free. The above models will be trained and validated for evaluation on two datasets of apples and persimmons, respectively, and the specific evaluation index results obtained are shown in **Table 3**. In addition, a picture with high detection difficulty is randomly selected in each of the two datasets and detected with the above models, respectively, and the detection effect images are shown in **Figures 9A, B**.

**Table 3:** Comparison results of detection of different models.

Network	Metric			
	AP/%	AP*^IOU^* ^=0.5/%^	AR/%	Time/ms
Apple Dataset				
FCOS	57.6	86.6	65.1	50.3
Faster-RCNN	59.2	85.9	65.1	54.5
YOLOv3	59.1	84.3	65.2	19.4
SSD	59.6	86.6	66.2	22.3
FSAF	61.7	87.6	68.5	54.2
ATSS	62.2	88.3	69.3	54.6
Ours	64.8	88.4	72.6	25.6
Persimmon Dataset				
FCOS	69.7	92.3	76.1	50.1
Faster-RCNN	70.7	91.3	76.1	54.3
YOLOv3	70.5	87.9	76.2	18.8
SSD	71.2	91.6	76.4	22.2
FSAF	72.1	92.1	78.1	54
ATSS	72.8	91.6	79.2	54.7
Ours	74.7	92	80.2	26.7

From the images of different model detection effects on the two datasets, it can be seen that some target fruits in the images that are not labeled because they are not easily labeled or forgotten at the time of labeling can basically be detected at the time of model detection, among which the detection effect of the model method in this paper is better. For the fruits that are severely obscured by leaves in the figure, several other models did not detect them, but this model can still detect them, and it can be seen that the detection accuracy of this model is higher compared with several other detection models in the case of overlapping obscured fruits with LED lighting at night.

From the comparison results of various evaluation indexes of different models shown in **Table 3**, it can be seen that the average detection accuracy of this model is better than several other detection models on two datasets, the average accuracy is 2.6-7.2 percentage points higher than other models on the apple dataset, and the average accuracy is 1.9-5 percentage points higher than other models on the persimmon dataset. For $AP^{IOU=0.5}$ and AR, the results of this model are also basically better than other models. In addition, the model results are most similar to the model in this paper for ATSS, and the average precision of the model in this paper is also 2.6% and 1.9% higher than ATSS on both datasets, and the average recall is 3.3% and 1% higher, respectively. When evaluating on the validation set, it is also necessary to consider the detection time for recognizing an image. Through **Table 3**, the average precision and average recall of FSAF and ATSS are closest to the results of the models in this paper, but the detection time used by the models in this paper to recognize an image is only about 45% of theirs. Overall, the model in this paper has a better real-time performance with higher average accuracy and average recall than the other models.

As can be seen from **Tables 3**, **4**, the model in this paper introduces some parameters, but the number of parameters is still lower than the anchor-based models Faster-RCNN and YOLOv3. The FLOPs and detection times of these two models are also higher than those of the model in this paper, and the average precision and average recall of the detection of the model in this paper on the green apple and green persimmon datasets are also significantly higher than those of these two models. In addition, compared with other models, the FLOPs of this model are only about 50% of those of the other models with some improvement in the average precision and recall rate.

**Table 4:** Comparison of assessment metrics of different detection models.

Network	Metric	
	FLOPs/G	Params/M
Faster-RCNN	206.66	41.12
YOLOv3	193.85	61.52
SSD	342.67	24.39
FSAF	202.39	36.01
ATSS	201.41	31.89
Ours	109.29	36.53

## 5 Conclusion

In order to improve the accuracy of fruit detection in modern orchards, this paper proposes an efficient target detection and recognition method with improved yolox-m. The model uses two datasets, unripe green persimmon and green apple, for training detection. Considering the complex situation of real orchards, the images collected in the dataset include leaf occlusion, fruit overlap and after rain. In this paper, we use Atrous Spatial Pyramid Pooling (ASPP) in the feature pyramid network to increase the receptive field and combine the feature information at different scales to improve the detection accuracy of the model, in addition, in order to mitigate the negative impact of sample imbalance and make the model focus more on positive samples to optimize the updated model parameters. For the loss function, the original binary cross-entropy (BCE) loss is replaced by varifocal loss to better optimize the model, improve the model performance and increase the precision.

The experimental results prove that the average precision, average recall and real-time performance of the model in this paper are better than those of several other models, and the computational complexity is also lower, which can achieve the detection and recognition of fruits accurately and in real time. It meets the needs of agricultural automation equipment. The model achieves a good level of detection on both datasets, however, it also has certain limitations, as follows:

(1) The number of images contained in the dataset used is relatively small due to realistic experimental conditions, and therefore we will consider continuing to expand the dataset.

(2) In order to improve the accuracy of the model, some parameters are introduced in this paper, and we will try to reduce the parameters of the model and reduce the size of the model in the future, while continuing to improve the accuracy.

## Data availability statement

The original contributions presented in the study are included in the article/supplementary material. Further inquiries can be directed to the corresponding authors.

## Author contributions

WJ, YX, and XG conceived the idea for the paper; YX, YL, and NP with contributions for data curation; YL and XY wrote the code, designed and conducted the experiments; YX and RJ with contributions for visualization and validation; WJ, YX, and XG with contributions for writing- original draft preparation. All authors contributed to the article and approved the submitted version.
